# Protective effect of dexmedetomidine on kidney injury of parturients with preeclampsia undergoing cesarean section: a randomized controlled study

**DOI:** 10.1042/BSR20190352

**Published:** 2019-05-03

**Authors:** Qing-lin Zhang, Lei Wang, Ming-jun Xu, Tian-long Wang

**Affiliations:** 1 Department of Anesthesiology, Xuanwu Hospital, Capital Medical University, Beijing 100053, P.R. China; 2 Gynaecological Oncology, Beijing Haidian Maternal & Child Health Hospital, Beijing 100080, P.R. China; 3 Department of Anesthesiology, Beijing Obsterics and Gynecology Hospital, Capital Medical University, Beijing 100020, P.R. China

**Keywords:** β2-MG, Dexmedetomidine, kidney injury, preeclampsia, SpO2, urine protein

## Abstract

The present study aimed to elucidate the effects of dexmedetomidine on kidney injury of parturients with preeclampsia (PE) undergoing cesarean section. Total 134 cesarean delivery women with PE were randomly divided into intervention group (IG) and control group (CG). Both groups underwent combined spinal and epidural anesthesia (CSEA), the IG was treated with 0.4 μg/(kg·min) dexmedetomidine for 10 min before surgery. The CG was treated with equivalent saline. Heart rate (HR), blood pressure, oxygen saturation (SpO_2_) of the two groups were measured at different time point after administration. Level of inflammatory factors were detected by enzyme-linked immunosorbent assay (ELISA). Visual analogue score (VAS), Ramsay sedation score (RSS), and kidney injury related indexes were evaluated at different time points. The plasma-drug concentration of patients was determined by High Performance Liquid Chromatography (HPLC) method. Compared with CG, HR, PE, and diastolic blood pressure (DBP) showed lower level while SpO_2_ showed higher level in IG. Furthermore, expression of tumor necrosis factor α (TNF-α), interleukin-6 (IL-6), and IL-10 in IG was decreased after drug administration, the contents of β2-MG, KIM-1 and urine protein were also decreased in contrast to the CG (all *P*<0.05). Besides, VAS score was decreased but Ramsay score was increased in the IG (both *P*<0.05). The results of HPLC showed that the half life of dexmedetomidine was about 20 min and it is speculated that the drug can be quickly metabolized within 24 h. Dexmedetomidine exerted protective effects on kidney injury of parturients with PE undergoing cesarean section.

## Introduction

As a hypertensive disorder, preeclampsia (PE) usually occurs on pregnant women. Individuals suffering from PE frequently exhibit hypertension, proteinuria as well as multiple systemic lesions. At the same time, PE may even cause fetal growth retardation, prematurity, still birth, and so on. [1]. Accumulating evidence indicates that PE often increases the degree of injury to the kidney and accelerates the rate of glomeruli and renal tubule dysfunction [2]. In addition, renal cortex and renal tubular necrosis caused by PE is confirmed to be an important inducement of acute kidney failure during pregnancy [3].

Dexmedetomidine is a highly selective α2 adrenergic receptor agonist commonly served in clinical practice, it has been proved to exert an active role in analgesia, sedation, and inhibition of sympathetic effects, thus widely applied to various kinds of operations [4]. A study performed by Eskandr et al. demonstrated that an appropriate amount of dexmedetomidine given during cesarean section under general anesthesia for pregnant women with PE can remarkably suppressed tracheal intubation response and hemodynamic response. At the same time, such method pose no negative impacts on the neonatal outcome [5]. A study which investigates the effect of dexmedetomidine on acute kidney injury caused by sepsis indicates that dexmedetomidine can decrease the apoptosis of renal tubular epithelial cells and local inflammatory response induced by lipopolysaccharide (LPS), suggesting a significant protective effect of dexmedetomidine on sepsis-induced acute kidney injury [6]. Another study designed by Leow et al. found that dexmedetomidine also exert protective an effect in renal injury caused by cardiac surgery in children [7]. However, the impact of dexmedetomidine on kidney injury of PE parturients remains largely unknown and warrants further investigation. Thus, we postulate that dexmedetomidine plays a protective role in PE and in the present study, we are aiming to test our hypothesis and offer sufficient supportive evidence for future therapeutic approach for kidney injury caused by PE.

## Materials and methods

### Ethic statement and trial registration

The present study was a prospective, randomized, double-blind, and controlled study. Ethical approval was obtained from the Ethics and Research Committee of the Department of Anesthesiology, Xuanwu Hospital of Capital Medical University. Informed consent had been obtained from all patients involved in the present study. The present study was also registered at Chinese Clinical Trial Registry and the registration number is ‘ChiCTR1800017726’.

### Study subjects

**Figure 1:**
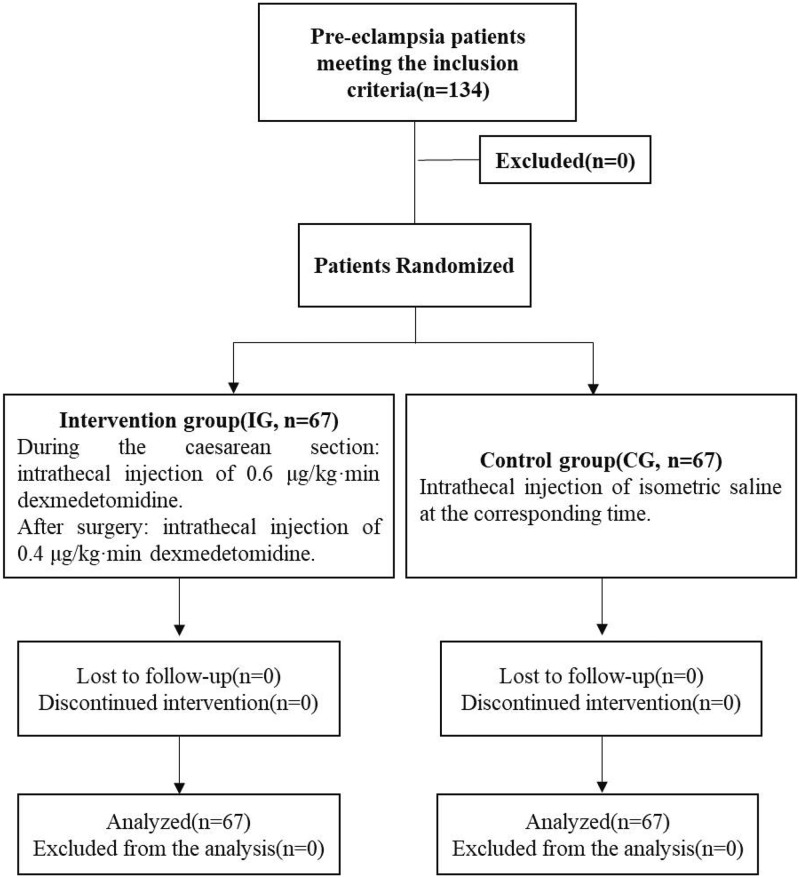
CONSORT flow diagram

The current study involved 134 PE patients undergoing caesarean section from June 2016 to July 2017. According to the American Society of Anesthesiologists physical status (ASA-PS) classification, patients were graded as ASA I-III, aged 25–39 years, with an average age of (29.8 ± 4.3) years, gestational weeks of 34–48 weeks, with an average gestational weeks of (36.3 ± 3.1) weeks, and body weights at 59–88 kg with an average of (69.3 ± 10.1) kg. The recruited patients were then randomly divided into intervention group (IG) and control group (CG) according to random number table, with 67 patients in each group. The patients received different treatments. Randomization was computer generated centrally by School of public health, capital medical university, Beijing, China and allocations were obtained by telephone or fax from staff who were not involved in other parts of the trial. Randomization was stratified by center with minimization for age, body weight, gestational weeks, and ASA grade. Patients and investigators were not masked to treatment assignment The details with regard to the participants have been shown in a Consolidated Standards of Reporting Trials (CONSORT) flow diagram (Figure 1).

### Inclusion and exclusion criteria

Diagnostic criteria [8]: PE is diagnosed by core symptoms of hypertension and proteinuria occurring after 20 weeks of pregnancy. The inclusion criteria were as following: patients met PE diagnostic criteria and cesarean section indications; 18–40 years old and the gestational age was above 34 weeks; systolic blood pressure (SBP) was above 140 but under 160 mmHg and the diastolic blood pressure (DBP) was above 90 but under 110 mmHg; the urinary protein dipstick was shown as + or ++. The exclusion criteria were as follows:patients who was allergic to dexmedetomidine; patients who have systemic diseases; patients who was treating with antipsychotic drugs or anticoagulant; patients who’s body mass index was above 35 [9].

### Anesthesia method and mode of administration [5]

Patients were fasted and taken in oxygen before surgery and 6% hydroxyethyl starch + 130/0.4 sodium chloride injection (H20103246, Fresenius Kabi, China) was administered prior to anesthesia. Patients were kept in left lateral position, CSEA was then performed in the spinal L3–L4 intervertebral space. Total 1 ml Ropivacaine Hydrochloride (H20140764, AstraZeneca AB, Sweden) diluted by 1 ml cerebrospinal fluid was injected into the patients’ spinal canal. The acupuncture method was utilized to determine the epidural block plane, and it was controlled in the T8–T10 segment of the spine, then the cesarean section was performed. Total 5 mg/(kg·h) propofol and 0.15 μg/(kg·min) remifentanil were injected in veins to retain anesthesia during operation. The bispectral index (BIS) was kept at 40–50 during operation.

The IG was intravenously and slowly injected by 0.4 μg/(kg·min) dexmedetomidine for 10 min before surgery (H20090248, Jiangsu Hengrui Pharmaceutical Co., Ltd., China), whereas the CG was injected with equal 0.9% normal saline for 10 min. Two anesthesiologists blinded to the experimental protocol were responsible for patient care and data collection during the surgery.

### Measurement of HR, SBP, DBP, and blood oxygen saturation

Levels of HR, SBP, DBP, and SpO_2_ of two groups of patients were measured by Philips MP60 Monitor (Philips, Holland), at 0 h (pre-operation), 1 h (postoperation), 3, 6, 12, and 24 h after administration.

### Enzyme-linked immuno sorbent assay

Total 3 ml venous blood of upper limb of the patients was drawn at 0 h (preoperation), 1 h (postoperation), 3, 6, 12, and 24 h after administration respectively. After standing for 15 min, serum samples were centrifugated at 3000 rpm/min for 15 min, then the supernatant was collected, and preserved in −80°C refrigerator. Levels of TNF-α, IL-6, and IL-10 were detected using TNF-α Quantikine ELISA Kit (DTA00C, Bio-Techne, U.S.A.), IL-6 Quantikine ELISA Kit (D6050, Bio-Techne, U.S.A.), IL-10 Quantikine ELISA Kit (D1000B, Bio-Techne, U.S.A.) respectively. The operating procedures were as following: first the reference standards (containing 50 μl reference standards) and samples (containing 10 μl sample under study + 40 μl sample dilution solutions) were constructed. Total 100 μl Horseradish Peroxidase (HRP)-conjugated antibody was added into the reference standards and samples, then the reaction pore was sealed off by closure plate membranes. The samples were bathed with water, which followed by the discard of liquid. Filled each well with washing solution and rest for 1 min. Discard the washing solution and dried the plate by using bilbulous paper, such washing procedure was repeated for five times. Total 50 μl substrates A and B were added to each well respectively and then the well was further incubated for 15 min in the dark. Subsequently, 50 μl stop solution was added into each well, a Microplate Reader (SpectraMax iD3, Molecular Devices, U.S.A.) was then applied for optical density (OD) detection at a value of 450 nm.

### Detection of renal function index

Urine of parturients was respectively collected at 0 d (preoperation), 1, 2, 3, 4, 5, 14, 21, and 42 d after the drug administration. The Automatic Biochemical Analyzer (P800, Roche, Switzerland) was utilized to detect the blood urea nitrogen (BUN), uric acid (UA), creatinine (Cr), β2-microglobulin (β2-MG), kidney injury molecule-1 (KIM-1), urine albumin (Alb) and total protein (TP) at various time points, the percentage of urine protein content was calculated.

### Detection of VAS and Ramsay Score [10]

Visual analogue score (VAS) and Ramsay sedation score (RSS) were used to evaluate the pain and sedation of the patients at 0 h (preoperation), 1 h (postoperation), 2, 3, and 4h after drug administration. The score of the VAS: 0 mark as painless, 10 mark as extremely painful. The score of the RSS: 1 mark as anxious, agitated, restless, 2 mark as cooperative, oriented, and tranquil, 3 mark as drowsiness and responsive to commands, 4 mark as sleeping and responsive to stimulus, 5 mark as sluggish response to stimulus, 6 mark as deep sleep and no response to stimulus.

### Determination of dexmedetomidine concentration in patients’ blood by **HPLC method [** 11]

High performance liquid chromatography (HPLC) assay was adopted to determine the blood concentration of PE treated with dexmedetomidine. The chromatographic condition was set up as following: Kromasil 60-5CN column was set as chromatographic column and the precolumn is Phenomenex Security Guard™ C_18_. Methanol: 0.5% formic acid solution (60:40) was set as mobile phase, the flow rate was 0.3 ml/min and column temperature was 35°C, volume of sample injection was 10 μl and the bottle temperature was 5°C. Sample treatment: 100 μl sodium hydroxide (1 mol/l) was added to 0.5 ml patient plasma, 20 μl dexmedetomidine (10 ng/ml) was set as internal standard. After mixing, 5 ml anhydrous ether was added, shaken for 3 min, and then centrifuged at 7000 rpm for 10 min. Discard the supernatant and the lower sediment was frozen at –80°C for 20 min. Subsequently, 150 μl mobile phase was added to dissolve the sediment. Then the solution was centrifuged at 1300 rpm for 10 min, 10 μl supernatant was collected and sampled. The determination of serum concentration was performed at 0, 10, 20, 30, 60, 120, 180, and 240 min after drug administration.

### Adverse events monitoring

The vital signs of the patients were monitored and recorded within 72 h after cesarean section. Major adverse reaction included bradycardia (<45 beats/min), hypotension (<60 mmHg), hypertension (>160 mmHg), localized pain, dry mouth, vomiting, and headache.

### Apgar score

The Apgar scores (activity, pulse, grimace, appearance, and respiration) of newborns in 1, 5, and 10 min were recorded after delivery. 8–10: no asphyxia, 4–7: mild asphyxia, 0–3: severe asphyxia.

### Statistical analysis

SPSS 21.0 software (IBM Corp., Armonk, NY, U.S.A.) was utilized for data analyses. Measurement data were expressed as mean ± S.D. The comparison between two groups was analyzed by *t* test, and multigroups were examined by one-way analysis of variance. (ANOVA). Enumeration data were expressed as a percentage and analyzed using a chi-square test, Comparisons amongst multiple groups were examined by ANOVA and homogeneity test of variance was performed, when there was a significant difference in variance analysis, *q* test was further conducted for pairwise comparison; in case of heteroscedasticity, non-parametric rank-sum was performed.

### Sample size analysis

The sample size analysis performed using Power Analysis and Sample Size 19.0.1 indicated that 42 patients were needed per group to detect a between-group difference with a power of 80%, α of 0.05, and an allocation ratio of 1:1 (based upon previous experience). So 67 patients in per group were included in the study.

## Results

### Demographics variables of the patients

The maternal and surgical characteristics of the two group patients were shown in Table 1 and detailed patient baseline characteristics were given in Supplementary Table S1. There was no significant difference in age, weight, height, gestational weeks, operation time, and haemorrhage between the two groups (*P*>0.05).

**Table 1:** The maternal and surgical characteristics of the patients

Group	N	Age (years old)	Weight (kg)	Height (cm)	Gestational age (week)	Operation time (min)	Amount of bleeding (ml)
IG	67	29.7 ± 3.1	62.8 ± 7.0	157.7 ± 3.4	38.0 ± 2.3	56.8 ± 6.9	275.9 ± 13.3
CG	67	29.0 ± 3.5	64.6 ± 5.6	158.8 ± 3.5	38.6 ± 2.8	58.4 ± 6.2	280.0 ± 12.6

Data are shown as mean ± S.D. CG, control group; IG, intervention group.

### Changes in basic vital signs of the parturients at different time points

As indicated in Table 2, no significant difference was observed in SBP, DBP, HR, and SpO_2_ value between the two groups at preoperative stage (both *P*>0.05). With the increase of time after injection, the values of SBP, DBP, and HR of the two groups were decreased, while the level of SpO_2_ was increased. Compared with the CG, the above indexes of the IG showed more obvious change (both *P*<0.05).

**Table 2:** Comparison of SBP, DBP, HR, and SpO_2_ at different time points between the two groups

Index	Group	n	0 h	1 h	3 h	6 h	12 h	24 h
SBP (mmHg)	IG	67	161.2 ± 10.2	155.2 ± 12.4*	133.3 ± 12.1*##	128.4 ± 13.0*##	126.5 ± 9.9*##	124.9 ± 11.6*##
	CG	67	162.0 ± 11.7	179.7 ± 10.1	175.8 ± 11.5#	174.8 ± 10.8#	174.4 ± 12.5#	172.4 ± 12.2#
DBP (mmHg)	IG	67	90.6 ± 9.1	99.5 ± 7.7	73.3 ± 6.7*##	74.2 ± 6.7*##	71.5 ± 6.0*##	70.5 ± 7.1*##
	CG	67	88.6 ± 7.8	101.3 ± 11.5	99.1 ± 11.5	98.4 ± 12.1	97.5 ± 12.9	97.0 ± 14.2#
HR (time / min)	IG	67	83.9 ± 8.5	88.3 ± 7.6*	70.0 ± 7.9*##	68.0 ± 5.2*##	64.6 ± 6.6*##	61.6 ± 4.4*##
	CG	67	83.2 ± 6.0	99.8 ± 10.4	98.7 ± 11.2	98.2 ± 9.6	97.0 ± 8.0	96.0 ± 12.4#
SpO_2_ (%)	IG	67	98.2 ± 6.6	86.7 ± 3.9	91.5 ± 6.7*#	93.5 ± 8.6*##	95.3 ± 5.6*##	97.7 ± 7.2*##
	CG	67	97.3 ± 8.3	88.1 ± 7.7	89.1 ± 5.7	90.1 ± 6.3	90.5 ± 7.3	91.9 ± 7.7#

Data are shown as mean ± S.D. *P<0.05, compared with the CG, and *#P*<0.05, *##P*<0.01, compared with 1 h.

### The inflammatory factor levels of the two groups at different time points

**Figure 2:**
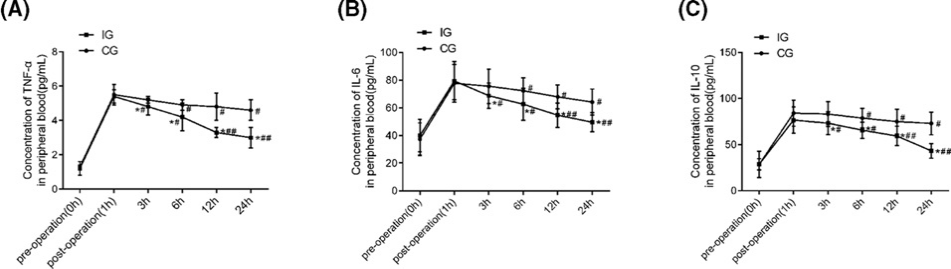
Comparison of concentrations of TNF-α, IL-6, and IL-10 in peripheral blood at different time points between the two groups (**A**) concentration of TNF-α in peripheral blood at different time points; (**B**) concentration of IL-6 in peripheral blood at different time points; (**C**) concentration of IL-10 in peripheral blood at different time points. In both groups, *n*=67. Compared with the CG, *P<0.05, **P<0.01; compared with 1 h, #*P*<0.05, ##*P*<0.01.

The levels of TNF-α, IL-6, and IL-10 in peripheral blood of the two groups at different time points were shown in Figure 2. The levels of TNF-α, IL-6, and IL-10 at pre-operation (0 h) and postoperation (1 h) of the two groups exhibited no significant difference (all *P*<0.05). With the increase of time after injection, levels of TNF-α, IL-6, and IL-10 showed a decline. Compared with the CG, IG showed the most obvious change (all *P*<0.05).

### Expression of kidney injury related indexes at different time points in the two groups

The expressions of kidney injury related indexes of the two groups at different time points were shown in Table 3. There was no significant difference in kidney injury-related indexs between two groups at preoperation (*P*>0.05). As time went on, the contents of Cr, UA, BUN, β2-MG, KIM-1, and urine protein were all declined, no significant difference was found in albumin and total protein content (*P*>0.05). Compared with the CG, the levels of β2-MG, KIM-1, and urine protein dropped more significantly at each time point after administration (*P*<0.05).

**Table 3:** Comparison of related indexes of kidney injury in urine between the two groups of patients at different time points

Index	Group	n	0 h	T1 d	T2 d	T3 d	T4 d	T5 d	T14 d	T21 d	T42d
Cr (μmol/l)	IG	67	57.3 ± 12.0	62.9 ± 13.4#	56.0 ± 7.7	55.0 ± 9.4	52.7 ± 12.7	52.6 ± 10.4#	52.8 ± 8.8#	52.0 ± 9.71#	52.4 ± 9.2#
	CG	67	55.9 ± 11.6	59.2 ±10.9	63.7 ± 11.1#	55.4 ± 9.4	53.0 ± 10.0	56.3 ± 7.1	55.5 ± 6.2	55.8 ± 5.8	56.3 ± 6.1
UA (UA, μmol/l)	IG	67	355.9 ± 74.7	353.0 ± 90.9	313.5 ± 41.8#	274.9 ± 90.4##	315.2 ± 83.7	286.7 ± 84.0#	283.5 ± 58.2#	287.8 ± 68.2#	283.5 ± 36.8#
	CG	67	347.5 ± 92.5	357.8 ± 70.1#	359.1 ± 97.1	353.5 ± 77.4	325.6 ± 90.3	312.8 ± 51.3	294.3 ± 91.9#	288.4 ± 57.1#	281.0 ± 38.7#
BUN (mmol/l)	IG	67	4.3 ± 1.8	4.4 ± 1.9	3.1 ± 1.2#	4.0 ± 1.4	4.2 ± 1.3	3.4 ± 1.0#	3.2 ± 0.5#	3.3 ± 1.1#	3.3 ± 1.0#
	CG	67	4.1 ± 1.5	4.2 ± 1.9	4.4 ± 2.0	4.2 ± 1.0	4.2 ± 1.2	3.2 ± 1.2#	3.2 ± 0.9#	3.2 ± 1.5#	3.1 ± 1.4#
KIM-1 (ng/l)	IG	67	4.6 ± 0.3	3.8 ± 0.5*#	3.4 ± 0.2**#	3.1 ± 0.1**#	2.9 ± 0.1**##	2.8 ± 0.2**##	2.6 ± 0.1**##	2.4 ± 0.2**##	2.2 ± 0.5**##
	CG	67	4.7 ± 0.2	4.4 ± 0.3	3.7 ± 0.3#	3.6 ± 0.5#	3.7 ± 0.4#	3.5 ± 0.5#	3.4 ± 0.2#	3.3 ± 0.4#	3.3 ± 0.7#
β2-MG (μg/l)	IG	67	1.73 ± 0.69	1.97 ± 0.65#	0.63 ± 0.27**##	0.31 ± 0.02**###	0.31 ± 0.02**###	0.31 ± 0.02###	-	-	-
	CG	67	1.67 ± 0.52	1.96 ± 0.49	1.45 ± 0.73#	0.67 ± 0.27##	0.65 ± 0.35##	0.30 ± 0.03####	-	-	-
Alb (mg/l)	IG	67	29.6 ± 5.6	27.9 ± 6.5	29.4 ± 10.0	29.8 ± 6.0	29.4 ± 5.6	30.4 ± 5.1	30.5 ± 5.7	30.4 ± 5.5	30.7 ± 2.6
	CG	67	30.8 ± 5.1	27.5 ± 6.6	26.2 ± 6.8	26.6 ± 5.3	27.0 ± 7.0	29.8 ± 5.1	29.4 ± 5.3	29.3 ± 4.8	29.4 ± 2.7
TP (mg/l)	IG	67	56.9 ± 6.8	55.5 ± 8.0	54.9 ± 10.9	56.8 ± 7.0	55.8 ± 6.7	59.4 ± 6.2	58.9 ± 5.4	59.1 ± 3.4	59.3 ± 3.1
	CG	67	54.7 ± 6.0	54.9 ± 9.3	51.7 ± 10.0	51.2 ± 10.6	52.1 ± 9.4	56.2 ± 9.0	57.0 ± 9.5	58.9 ± 5.4	59.3 ± 4.6
Urinary protein ratio (%)	IG	67	100	100	93.1*#	83.2*#	53.4**##	53.7*##	16.7**###	-	-
	CG	67	100	100	100	96.8	96.4	76.7##	42.8##	9.9###	3.3###

Data are shown as mean ± S.D. or number (percentage).β2-MG, β2-microglobulin. *P<0.05, **P<0.01, ***P<0.001, compared with the CG, and #*P*<0.05, *##P*<0.01, ### *P*<0.001, compared with 0 h. The concentration of index detected is below the threshold.

### Comparison of VAS and RSS between the two groups at different time points

The VAS and RSS of the two groups of parturients were shown in Table 4. There was no statistical difference in VAS and RSS between the two groups at preoperation (both *P*>0.05). In IG, VAS was gradually decreased with the increase of time after drug injection, but the RSS was gradually increased (both *P<*0.05), whereas VAS and RSS in the CG were fluctuating. Compared with the CG, the VAS of the IG was decreased at each time point after drug administration, but the RSS was increased (all *P*<0.05).

**Table 4:** Comparison of VAS and RSS between the two groups of patients at different time points

Index	Group	n	0 h	1 h	2 h	3 h	4 h
VAS	IG	67	4.2 ± 0.9	6.3 ± 0.8*	4.8 ± 0.9*##	3.5 ± 0.6*##	2.1 ± 0.6*##
	CG	67	4.4 ± 0.7	7.5 ± 0.6	6.9 ± 0.4	7.4 ± 0.4	7.1 ± 0.5
RSS	IG	67	3.4 ± 0.4	2.1 ± 0.6	2.7 ± 0.4*#	4.2 ± 0.7*##	4.9 ± 0.7*##
	CG	67	3.3 ± 0.4	2.1 ± 0.4	1.9 ± 0.4	2.2 ± 0.6	2.0 ± 0.4

Data are shown as mean ± S.D. RSS, Ramsay sedation score; VAS, visual analogue score. *P<0.05, compared with the CG, and *#P*<0.05, *##P*<0.01 when compared with 1 h.

### Blood concentration of the parturients treated with dexmedetomidine at different time points

**Figure 3:**
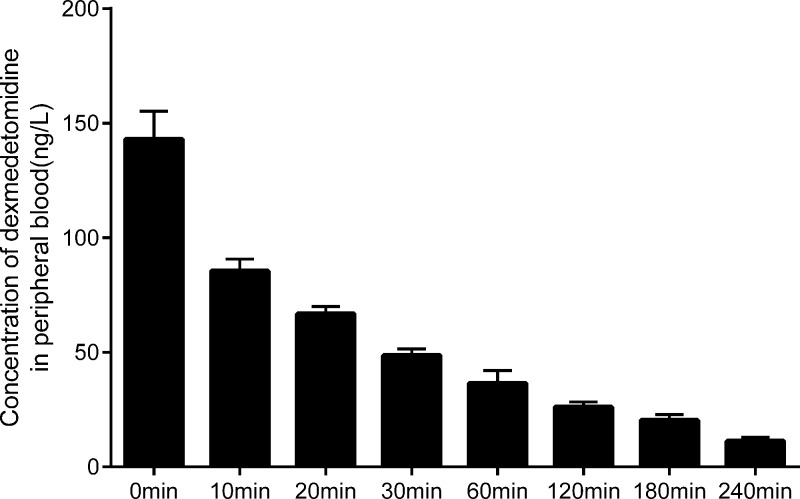
Blood concentrations of dexmedetomidine at different time points (ng/L).

HPLC method was utilized to detect the concentration of dexmedetomidine in blood of the IG at different time points. As indicated in the (Figure 3), the concentration of dexmedetomidine in the patients’ blood was reduced by half after 20 minutes of drug injection. Moreover, after 240 minutes of injection, it was reduced to about 1/10 of the original. The results revealed that dexmedetomidine experienced a faster metabolism in cesarean section of PE parturient, and its efficacy can be effectively exerted.

### Drug adverse reaction monitoring in two groups

The incidence of adverse drug reactions in patients 72 h after cesarean section and Apgar scores of newborns in 1, 5, and 10 min were shown in Tables 5 and 6. There was a higher proportion of bradycardia and hypotension in IG compared with the CG (both *P*<0.05). After relevant measures, the patients were improved and returned to normal. In addition, Apgar scores of neonates within 1, 5, and 10 min after delivery were not significantly different between two groups(both *P*<0.05).

**Table 5:** Adverse drug reactions in two groups of patients

Group	n	Bradycardia	Hypotension	Hypertension	Local pain	Dry mouth	Vomiting	Headache
IG	67	8 (26.7%)*	7 (23.3%)**	2 (6.7%)*	1 (3.3%)	5 (16.7%)	0 (0%)	0 (0%)
CG	67	2 (6.7%)	1 (3.3%)	6 (20%)	2 (6.7%)	4 (13.3%)	2 (6.7%)	0 (0%)

Data are shown as number (percentage). CG, control group; IG, intervention group. *P<0.05, **P<0.01 when compared with the CG.

**Table 6:** Apgar scores in two groups of newborns

Group	n	1min	5min	10min
IG	67	9.3±0.5	9.4±0.6	9.6±0.2
CG	67	9.1±0.4	9.5±0.3	9.5±0.7

Data are shown as mean ± S.D..

## Discussion

A study designed by Awowole IO demonstrated that 40% of kidney damage of parturients was associated with PE [2]. Patients with PE are usually accompanied by vasospasm and pachemia, which can further lead to pathological changes such as reduced kidney blood perfusion or kidney tubular necrosis. In case of other complications during pregnancy at the same time, parturients are prone to develop acute kidney injury,which will become life threatening if severe [12]. Therefore, it is of a pivotal importance to find a safe and effective therapeutic method for kidney injury of PE parturients, so as to improve the pregnancy outcome. The PE patients were treated undergoing cesarean section with dexmedetomidine in present study and the results indicated that dexmedetomidine can improve the hypertension, relieve pain as well as strengthen calmness, furthermore, it also displayed a significant protective effect on kidney injury of parturients.

It has been confirmed that dexmedetomidine exerts notable positive effects in inhibiting plasma norepinephrine levels and inhibiting sympathetic nerves; thus, it was widely used in perioperative analgesia and sedation for various types of surgeries [13]. In recent years, accumulating evidence illustrates that dexmedetomidine also plays a protective role in kidney damage caused by sepsis and cardiac surgery [14, 15]. The possible mechanism for its protection against kidney damage induced by the above-mentioned diseases may be that dexmedetomidine can inhibit the sympathetic tension, decrease the inflammatory response and renal ischemia-reperfusion injury through binding to α2 adrenergic receptors [16]. Another study indicate that the dexmedetomidine can down-regulate the apoptosis of kidney tubular epithelial cells by inhibiting activation of signaling pathways such as Janus kinase/signal transducer and activator of tran-ions (JAK/STAT) and mitogen-activated protein kinase (MAPK) [17]. The present study demonstrates the value of dexmedetomidine in the protection of cesarean section of women with PE for the first time. Patients in the IG displayed significantly lower levels of SBP, DBP, and HR and increased levels of SpO_2_ after surgery compared with the CG (all *P<*0.05), suggesting the value of dexmedetomidine in effectively improving hypertension of PE parturients. This finding is also confirmed by the research conducted by Hariharan et al [18]. Dexmedetomidine exerts an analgesic effect primarily because it can inhibit peripheral C fibers and Aα fibers [19]. In addition, it can also act on the α2 receptor in the locus and the synaptic membrane of the spinal cord, subsequently inhibit the transmission of noxious stimuli and pain signals to the brain [20]. At the same time, dexmedetomidine can offer anxiolytic, hypnotic and sedative effects on patients through acting on the α2A receptor in the blue spot [21]. In the present study, PE parturients treated with intrathecal injection of dexmedetomidine displayed a significantly lower VAS and higher RSS after drug administration conpared with the CG further confirmed the superiority of dexmedetomidine in analgesia and sedation.

Cr and urinary protein contents are critical indicators for clinical diagnosis of kidney injury and their enhancements in expression are considered to be closely correlated to the extent of renal trauma [22]. In addition, another study attested that the levels of urinary KIM-1 and β2-MG in PE parturients were significantly higher than those in normal parturients, suggesting that the level of β2-MG and KIM-1 in the urine play a vital role in preeclamptic kidney damage [23]. Liang *et al.* applied dexmedetomidine in cisplatin-based cancer chemotherapy and it was found that apart from promote to keep calm and dexmedetomidine can also exert protective effects in kidney damage induced by cisplatin. The regulation of apoptosis and immune responses may involved in the underlying mechanisms [24]. In the present study, though no significant difference was observed in kidney function indexes between the two groups before operation, the levels of β2-MG, KIM-1, and urinary protein in the IG were significantly lower than those in the CG at each time point after drug administration (*P*<0.05), indicating that dexmedetomidine plays a protective role in improving kidney injury of PE parturients during cesarean section. Futhermore, the response of an overall pro-inflammatory systemic environment is verified to be closely related to the morbidity of PE, the excessive release of inflammatory factors could contribute to the progression of PE, heart and kidney damage of patients [25]. In present study, the level of inflammatory factors in peripheral blood of patients in IG was significantly lower than that in the CG, demonstrating that dexmedetomidine is beneficial for inhibiting the release of inflammatory factors, the protective effects of dexmedetomidine against kidney damage in PE may be achieved by inhibiting the maternal inflammatory response and thereby reducing the pathological damage caused by inflammatory factors to the kidney. However, whether the immunomodulatory effects of dexmedetomidine work through other signal pathway or affected by other factors still remains unknown; thus, a larger number of experiments should be conducted in the future to further elucidate the underlying mechanism of dexmedetomidine in kidney injury of PE parturients undergoing cesarean section.

In conclusion, the aforementioned findings of the study strongly support the standpoint that dexmedetomidine cannot only promote analgesia and sedation, alleviate the symptoms of PE, but also contributes to down-regulating the levels of β2-MG, KIM-1, and urine protein, exerting a significant protective effect on kidney injury in PE parturients. Our study is of guiding significance in medicine application for PE parturients undergoing cesarean section.

## Ethic Statements

The study was in accordance with the Helsinki Declaration and it was discussed and approved by the Ethics Committee of the Xuanwu Hospital of Capital Medical University. Patients volunteered to participate and signed informed consent forms. The trial was registered at Chictr.org.cn (ID ChiCTR1800017726).

## Supporting information

**Supplemental Table S1:** 

## Abbreviations

Alb: albumin
BUN: blood urea nitrogen
CG: control group
CONSORT: Consolidated Standards of Reporting Trial
Cr: creatinine
CSEA: combined spinal and epidural anesthesia
DBP: diastolic blood pressure
HR: heart rate
IG: intervention group
KIM-1: kidney injury molecule-1
MAPK: mitogen-activated protein kinase
PE: preeclampsia
RSS: Ramsay sedation score
SBP: systolic blood pressure
TNF-α: tumor necrosis factor α
TP: total protein
UA: uric acid
VAS: visual analogue score
β2-MG: β2-microglobulin

## References

[1] Xu Y., Wu D., Jiang Z., Zhang Y., Wang S., Ma Z. (2018) MiR-616-3p modulates cell proliferation and migration through targeting tissue factor pathway inhibitor 2 in preeclampsia. Cell Prolif. e12490 10.1111/cpr.12490 30028057 PMC6528919

[2] Awowole I.O., Omitinde O.S., Arogundade F.A., Bola-Oyebamiji S.B. and Adeniyi O.A. (2018) Pregnancy-related acute kidney injury requiring dialysis as an indicator of severe adverse maternal morbidity at a tertiary center in Southwest Nigeria. Eur. J. Obstet. Gynecol. Reprod. Biol. 225, 205–209 10.1016/j.ejogrb.2018.04.041 29751278

[3] Park S.K., Hur M. and Kim W.H. (2018) Acute kidney injury in parturients with severe preeclampsia. J. Anesth. 10.1007/s00540-018-2535-3 30043102

[4] Ibrahim E., Sultan W., Helal S., Abo-Elwafa H. and Abdelaziz A. (2018) Pregabalin and dexmedetomidine conscious sedation for flexible bronchoscopy: a randomized double blind controlled study. Minerva Anestesiol. 85, 487–493 10.23736/S0375-9393.18.12685-X 30021408

[5] Eskandr A.M., Metwally A.A., Ahmed A.A., Elfeky E.M., Eldesoky I.M., Obada M.A. (2018) Dexmedetomidine as a part of general anaesthesia for caesarean delivery in patients with pre-eclampsia: A randomised double-blinded trial. Eur. J. Anaesthesiol. 35, 372–378 29432379 10.1097/EJA.0000000000000776

[6] Kang K., Gao Y., Wang S.C., Liu H.T., Kong W.L., Zhang X. (2018) Dexmedetomidine protects against lipopolysaccharide-induced sepsis-associated acute kidney injury via an alpha7 nAChR-dependent pathway. Biomed. Pharmacother. 106, 210–216 10.1016/j.biopha.2018.06.059 29960167

[7] Leow E.H., Chan Y.H., Ng Y.H., Lim J. K.B., Nakao M. and Lee J.H. (2018) Prevention of acute kidney injury in children undergoing cardiac surgery: a narrative review. World J. Pediatr. Congenit. Heart Surg. 9, 79–90 10.1177/2150135117743211 29310552

[8] Redman C.W. and Roberts J.M. (1993) Management of pre-eclampsia. Lancet 341, 1451–1454 10.1016/0140-6736(93)90890-S 8099149

[9] Yousef A.A., Salem H.A. and Moustafa M.Z. (2015) Effect of mini-dose epidural dexmedetomidine in elective cesarean section using combined spinal-epidural anesthesia: a randomized double-blinded controlled study. J. Anesth. 29, 708–714 10.1007/s00540-015-2027-7 26006222

[10] Wang X., Liu W., Xu Z., Wang F., Zhang C., Wang B. (2016) Effect of dexmedetomidine alone for intravenous patient-controlled analgesia after gynecological laparoscopic surgery: a consort-prospective, randomized, controlled trial. Medicine (Baltimore) 95, e3639 10.1097/MD.0000000000003639 27175680 PMC4902522

[11] Szerkus O., Struck-Lewicka W., Kordalewska M., Bartosinska E., Bujak R., Borsuk A. (2017) HPLC-MS/MS method for dexmedetomidine quantification with Design of Experiments approach: application to pediatric pharmacokinetic study. Bioanalysis 9, 395–406 10.4155/bio-2016-0242 28105858

[12] Pinton L., Solito S., Masetto E., Vettore M., Cane S., Puppa A.D. (2018) Immunosuppressive activity of tumor-infiltrating myeloid cells in patients with meningioma. Oncoimmunology 7, e1440931 10.1080/2162402X.2018.1440931 29900047 PMC5993508

[13] Farghaly H.S., Mahmoud A.M. and Abdel-Sater K.A. (2016) Effect of dexmedetomidine and cold stress in a rat model of neuropathic pain: Role of interleukin-6 and tumor necrosis factor-alpha. Eur. J. Pharmacol. 776, 139–145 10.1016/j.ejphar.2016.02.046 26896779

[14] Ma S., Evans R.G., Iguchi N., Tare M., Parkington H.C., Bellomo R. (2018) Sepsis-induced acute kidney injury: a disease of the microcirculation. Microcirculation e12483 29908046 10.1111/micc.12483

[15] Man J., Ritchie G., Links M., Lord S. and Lee C.K. (2018) Treatment-related toxicities of immune checkpoint inhibitors in advanced cancers: a meta-analysis. Asia Pac. J. Clin. Oncol. 14, 141–152 10.1111/ajco.12838 29349927

[16] Liu Y.E., Tong C.C., Zhang Y.B., Jin H.X., Gao Y. and Hou M.X. (2015) Effect of dexmedetomidine on rats with renal ischemia-reperfusion injury and the expression of tight junction protein in kidney. Int. J. Clin. Exp. Med. 8, 18751–18757 26770491 PMC4694391

[17] Sutepvarnon A., Warnnissorn M. and Srimuninnimit V. (2013) Predictive value of Ki67 for adjuvant chemotherapy in node-negative, hormone receptor-positive breast cancer. J. Med. Assoc. Thai. 96, S60–S66 23590023

[18] Hariharan U. (2017) Postpartum hemorrhage and pregnancy induced hypertension during emergency lower segment cesarean section: dexmedetomidine to our rescue. Rev. Bras. Anestesiol. 67, 538–540 10.1016/j.bjan.2015.09.001 28535940

[19] Park H.J., Kim Y.H., Koh H.J., Park C.S., Kang S.H., Choi J.H. (2012) Analgesic effects of dexmedetomidine in vincristine-evoked painful neuropathic rats. J. Korean Med. Sci. 27, 1411–1417 10.3346/jkms.2012.27.11.1411 23166426 PMC3492679

[20] Fukuda M., Vazquez A.L., Zong X. and Kim S.G. (2013) Effects of the alpha(2)-adrenergic receptor agonist dexmedetomidine on neural, vascular and BOLD fMRI responses in the somatosensory cortex. Eur. J. Neurosci. 37, 80–95 10.1111/ejn.12024 23106361 PMC3538949

[21] Ciftci T., Erbatur S. and Ak M. (2015) Comparison of the effects of dexmedetomidine and remifentanil on potential extreme haemodynamic and respiratory response following mask ventilation and laryngoscopy in patients with mandibular fractures. Eur. Rev. Med. Pharmacol. Sci. 19, 4427–4433 26636533

[22] Vanmassenhove J., Vanholder R., Nagler E. and Van Biesen W. (2013) Urinary and serum biomarkers for the diagnosis of acute kidney injury: an in-depth review of the literature. Nephrol. Dial. Transplant. 28, 254–273 10.1093/ndt/gfs380 23115326

[23] Codsi E., Garovic V.D., Gonzalez-Suarez M.L., Milic N., Borowski K.S., Rose C.H. (2017) Longitudinal characterization of renal proximal tubular markers in normotensive and preeclamptic pregnancies. Am. J. Physiol. Regul. Integr. Comp. Physiol. 312, R773–R778 10.1152/ajpregu.00509.2016 28438765

[24] Liang H., Liu H.Z., Wang H.B., Zhong J.Y., Yang C.X. and Zhang B. (2017) Dexmedetomidine protects against cisplatin-induced acute kidney injury in mice through regulating apoptosis and inflammation. Inflamm. Res. 66, 399–411 10.1007/s00011-017-1023-9 28224201

[25] Szarka A., Rigo J. Jr, Lazar L., Beko G. and Molvarec A. (2010) Circulating cytokines, chemokines and adhesion molecules in normal pregnancy and preeclampsia determined by multiplex suspension array. BMC Immunol. 11, 59 10.1186/1471-2172-11-59 21126355 PMC3014878

